# Supplementary material for: Antimalarial and neuroprotective effects of ethanolic extracts of the five-flower remedy in an experimental cerebral malaria model
Source: PLoS One. 2025 Sep 2;20(9):e0330880. doi: 10.1371/journal.pone.0330880 (PMC12404382; doi:10.1371/journal.pone.0330880)
Supplement: S1 File — (PDF) [file pone.0330880.s001.pdf]

Supporting information file 1

Data tables for graph generation

Table 1 Effects of crude extract from the five-flower remedy on percentage of parasite in an experimental cerebral malaria at day 4, 5, 6, 7, 8, 10, 12, and 13 post-infections. (Data presented corresponds to Figure 2)

	***Pb*A infected**	**Art**	**FFR**	**Art + FFR**
**Day 4**	6.88 ± 0.58	6.61 ± 0.26	4.96 ± 0.37	6.17 ± 0.35
**Day 5**	7.50 ± 0.83	10.55 ± 0.11	11.77 ± 1.05	10.74 ± 2.08
**Day 6**	15.06 ± 1.45	16.60 ± 0.37	15.72 ± 0.45	15.15 ± 0.57
**Day 7**	21. 53 ± 0.44	22.16 ± 1.21	19.11 ± 2.46	19.96 ± 0.96
**Day 8**	24.00 ± 0.64$^{b, c, d}$	18.20 ± 0.85$^{a, d}$	20.05 ± 0.61$^{a, d}$	15.20 ± 0.12$^{a, b, c}$
**Day 10**	30.44 ± 0.68 $^{b, c, d}$	15.94 ± 1.09^{a}	18.84 ± 0.73$^{a, d}$	13.68 ± 0.47$^{a, c}$
**Day 12**	38.53 ± 1.27 $^{b, c, d}$	14.88 ± 0.28$^{a, d}$	16.22 ± 0.33$^{a, d}$	11.15 ± 0.36$^{a, b, c}$
**Day 13**	44.47 ± 2.49 $^{b, c, d}$	14.68 ± 0.87 $^{a, d}$	15.91 ± 1.02 $^{a, d}$	9.81± 0.42$^{a, b, c}$

Data are expressed as the mean ± SEM (n= 5/group), $p < 0.05$. [a]Significantly higher than that of the negative control. [b]Significantly higher than those of the groups treated with artesunate. [c]Significantly higher than those of the groups treated with FFR 600 mg/kg extract. [d]Significantly higher than those of the groups treated with Art + FFR 600 mg/kg extract.

Table 2 Assessment of crude extracts from the Five flower Remedy in an experimental cerebral malaria model. Clinical scores were recorded on days 1, 4, 5, 6, 8, 10, and 12 post-infections. (Data presented corresponds to Figure 3)

	**Control uninfected**	***Pb*A infected**	**Art**	**FFR**	**Art + FFR**
**Day 1**	20.00 ± 0.00	20.00 ± 0.00	20.00 ± 0.00	20.00 ± 0.00	20.00 ± 0.00
**Day 4**	20.00 ± 0.00$^{b, c, d, e}$	16.00 ± 0.31^{a}	15.40 ± 0.24^{a}	15.60 ± 0.24^{a}	15.40 ± 0.24^{a}
**Day 5**	20.00 ± 0.00 $^{b, c, d, e}$	15.40 ± 0.24^{a}	14.60 ± 0.24^{a}	14.60 ± 0.24^{a}	14.40 ± 0.24^{a}
**Day 6**	20.00 ± 0.00 $^{b, c, d, e}$	14.00 ± 0.31^{a}	13.20 ± 0.37^{a}	13.60 ± 0.40^{a}	13.00 ± 0.31^{a}

**Day 8**	20.00 ± 0.00 [b, c, d, e]	11.20 ± 0.37[a, e]	12.20 ± 0.48[a]	11.80 ± 0.73[a]	13.80 ± 0.48[a, b]
**Day 10**	20.00 ± 0.00 [b, c, d, e]	10.60 ± 0.24[a, c, d, e]	13.20 ± 0.48[a, b]	13.00 ± 0.94[a, b, e]	15.20 ± 0.37[a, b, d]
**Day 12**	20.00 ± 0.00 [b, c, d, e]	9.60 ± 0.24 [a, c, d, e]	14.00 ± 0.31[a, b, e]	14.00 ± 0.54[a, b, e]	16.20 ± 0.37 [a, b, c, d]

Data are expressed as the mean ± SEM (n= 5/group), $p < 0.05$. [a]Significantly higher than that of the control uninfected. [b]Significantly higher than that of the negative control. [c]Significantly higher than those of the groups treated with artesunate. [d]Significantly higher than those of the groups treated with 600 mg/kg extract. [e]Significantly higher than those of the groups treated with Art+600 mg/kg extract.

Table 3 Effect of crude extracts from the Five flower Remedy on cognitive dysfunction and long-term memory impairment in experimental cerebral malaria model using novel object recognition test. (Data presented corresponds to Figure 4)

Groups of treatment	% Discrimination
**Control uninfected**	35.18 ± 1.65[b, c, d]
***Pb*A infected**	14.97 ± 2.81[a, d, e]
**Art**	22.03 ± 2.42[a, d]
**FFR**	25.86 ± 2.42[a, b, d]
**Art + FFR**	35.86 ± 1.13[b, c, d]

Data are expressed as the mean ± SEM (n= 5/group), $p < 0.05$. [a]Significantly higher than that of the control uninfected. [b]Significantly higher than that of the negative control. [c]Significantly higher than those of the groups treated with artesunate. [d]Significantly higher than those of the groups treated with 600 mg/kg extract. [e]Significantly higher than those of the groups treated with Art+600 mg/kg extract.

Table 4 Comparative analysis of the effects of Five-Flower Remedy crude extract on the expression of inflammatory cytokines and neurotrophic factors in the brain of *Pb*A-infected mice. (Data presented corresponds to Figure 5)

	**Control uninfected**	***Pb*A infected**	**Art**	**FFR**	**Art + FFR**
**TNF-α**	1.00 ± 0.00[b, c, d]	2.34 ± 0.16[a, e]	2.25 ± 0.15[a, e]	1.80 ± 0.03[a]	1.18 ± 0.12[b, c]
**IL-1β**	1.00 ± 0.00[b]	1.78 ± 0.14[a, c, d, e]	1.20 ± 0.01[b]	1.17 ± 0.05[b]	1.10 ± 0.08[b]
**BDNF**	1.00 ± 0.00[c, d, e]	0.91 ± 0.01[c, d, e]	8.32 ± 1.39[a, b d, e]	12.63 ± 0.69[a, b, c]	13.35 ± 0.67[a, b, c]
**Trk B**	1.00 ± 0.00[c, d, e]	0.88 ± 0.08[c, d, e]	13.29 ± 2.75[a, b]	16.26 ± 2.34[a, b]	19.18 ± 0.63[a, b]

Data are expressed as the mean ± SEM (n= 5/group), $p < 0.05$. [a]Significantly higher than that of the control uninfected. [b]Significantly higher than that of the negative control. [c]Significantly higher than those of the groups treated with artesunate. [d]Significantly higher than those of the groups treated with 600 mg/kg extract. [e]Significantly higher than those of the groups treated with Art+600 mg/kg extract.
