# Supplementary material for: Antimalarial and neuroprotective effects of ethanolic extracts of the five-flower remedy in an experimental cerebral malaria model
Source: PLoS One. 2025 Sep 2;20(9):e0330880. doi: 10.1371/journal.pone.0330880 (PMC12404382; doi:10.1371/journal.pone.0330880)
Supplement: S2 File — (PDF) [file pone.0330880.s002.pdf]

Supporting information file 2

Brain histopathology in an experimental cerebral malaria (ECM) model

(uncropped raw images)

Uninfected control

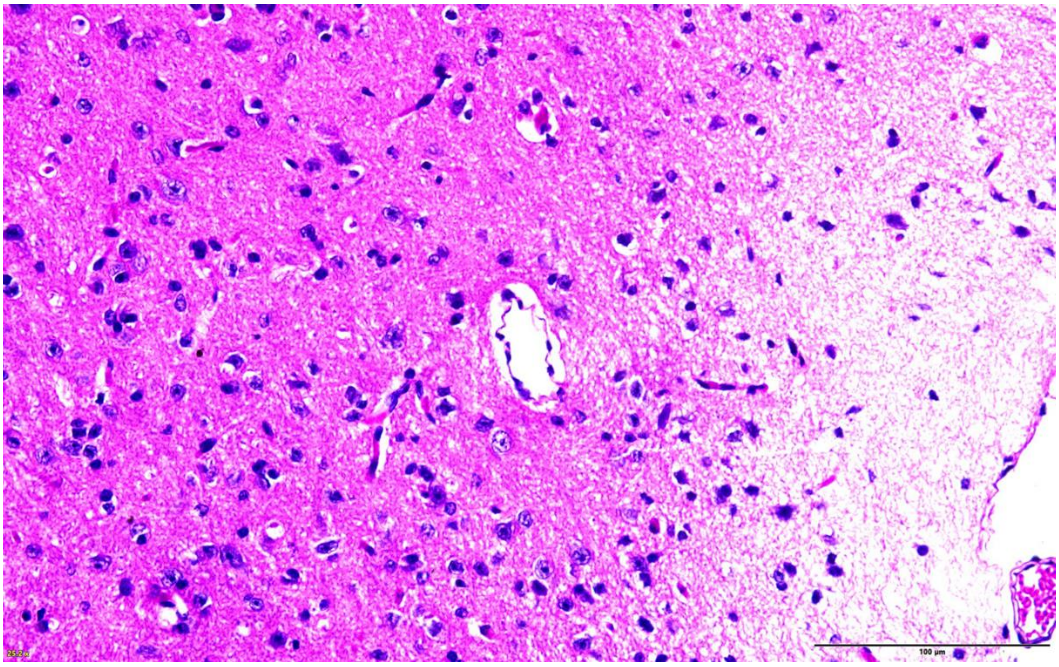

6A. Blood vessels

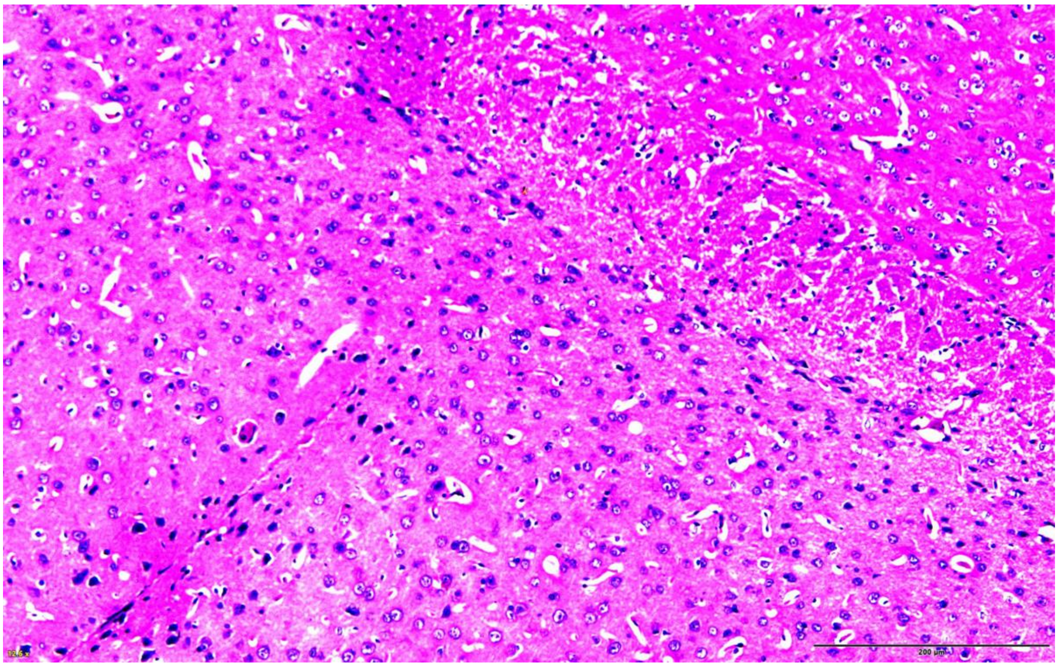

6B. Brain tissue

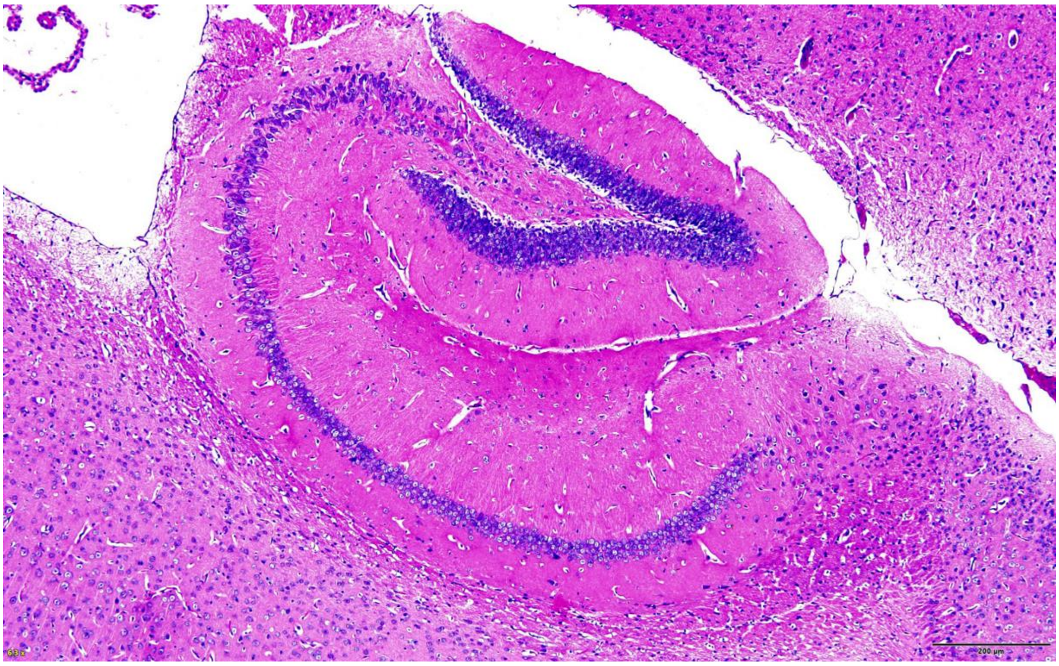

6C. Region of the hippocampus

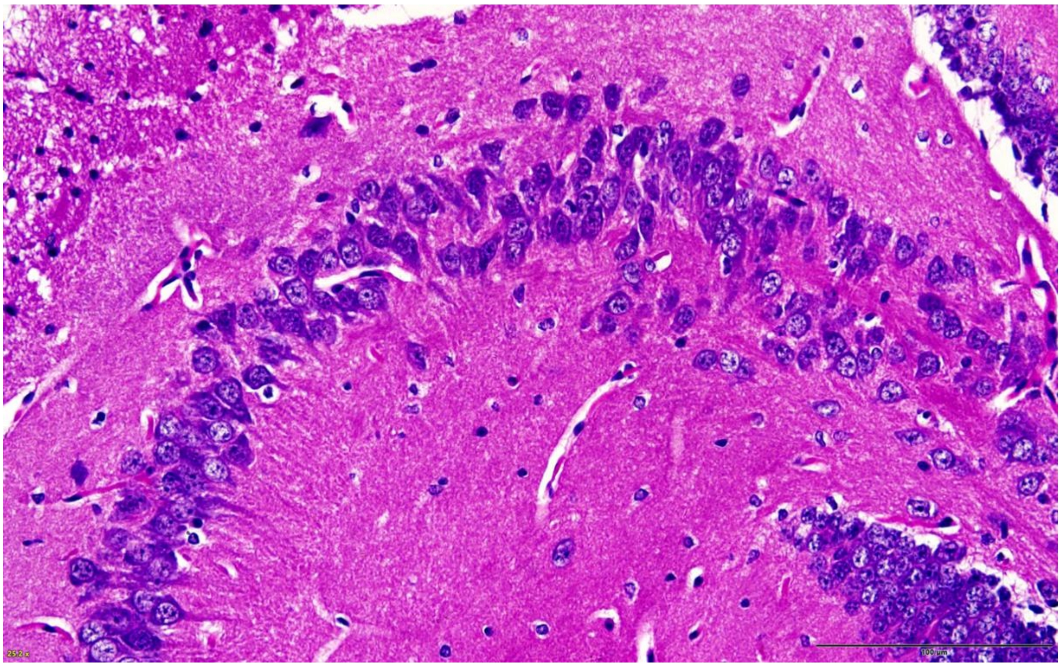

6C. Region of the hippocampus (black box)

Plasmodium berghei ANKA (*PbA*)

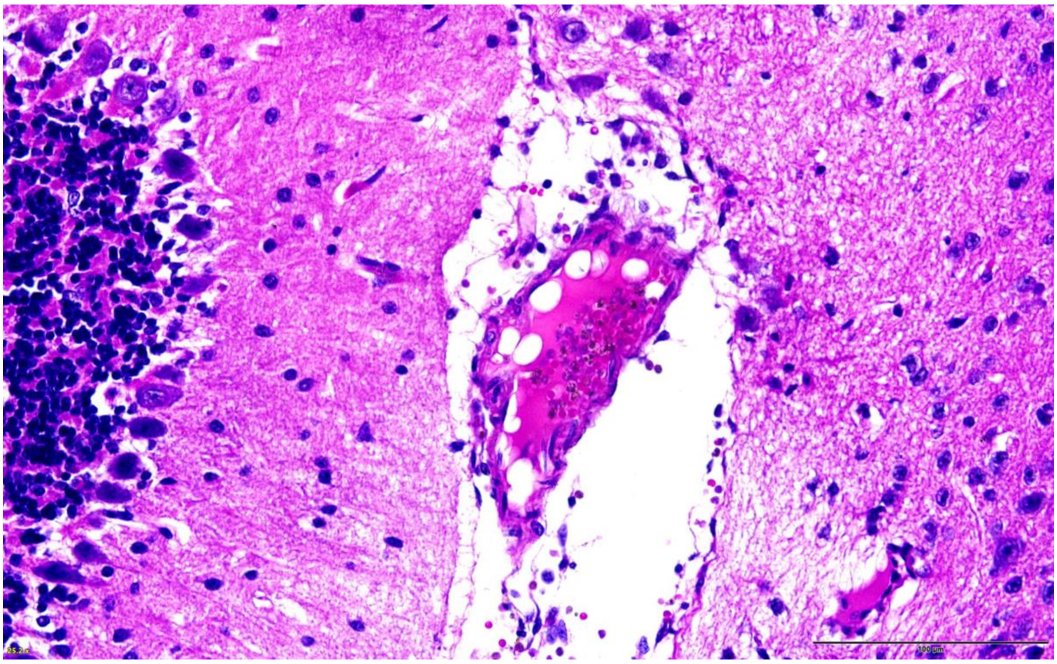

6D. Blood vessels

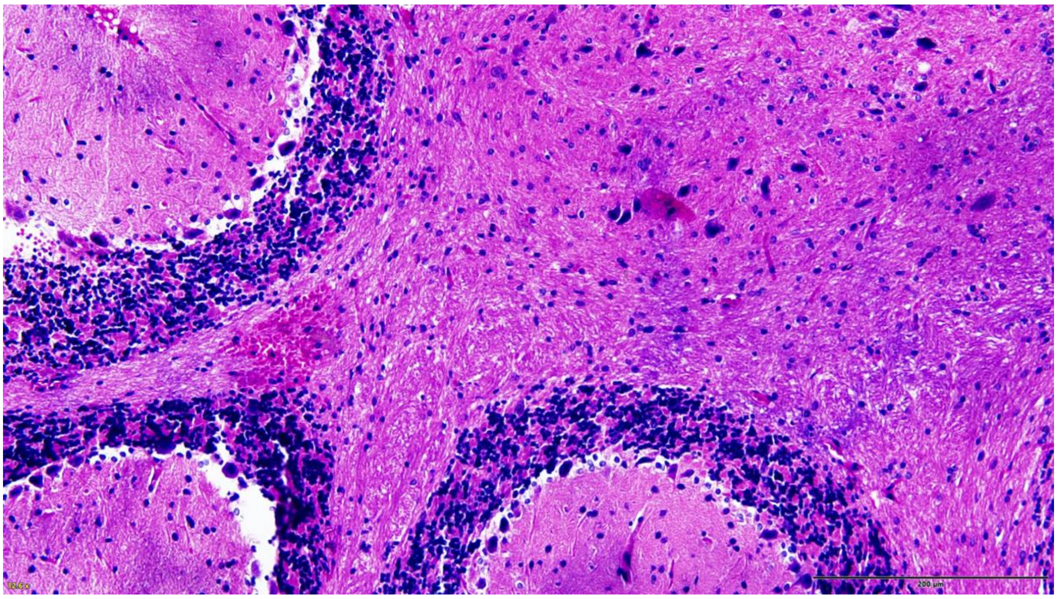

6E. Brain hemorrhage

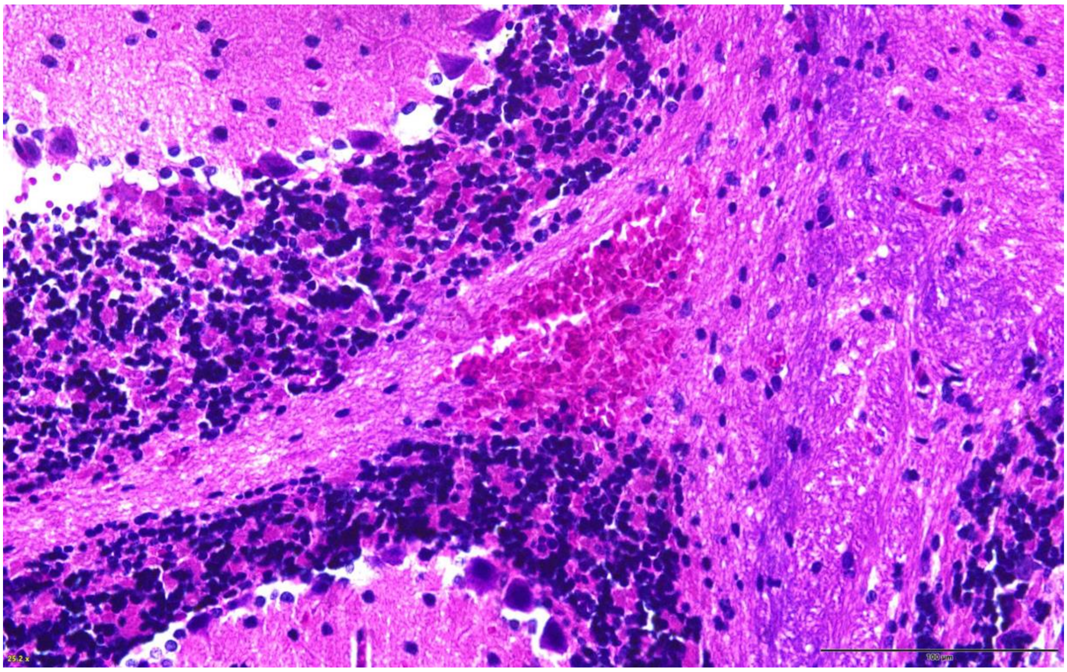

6E. Brain hemorrhage (black box)

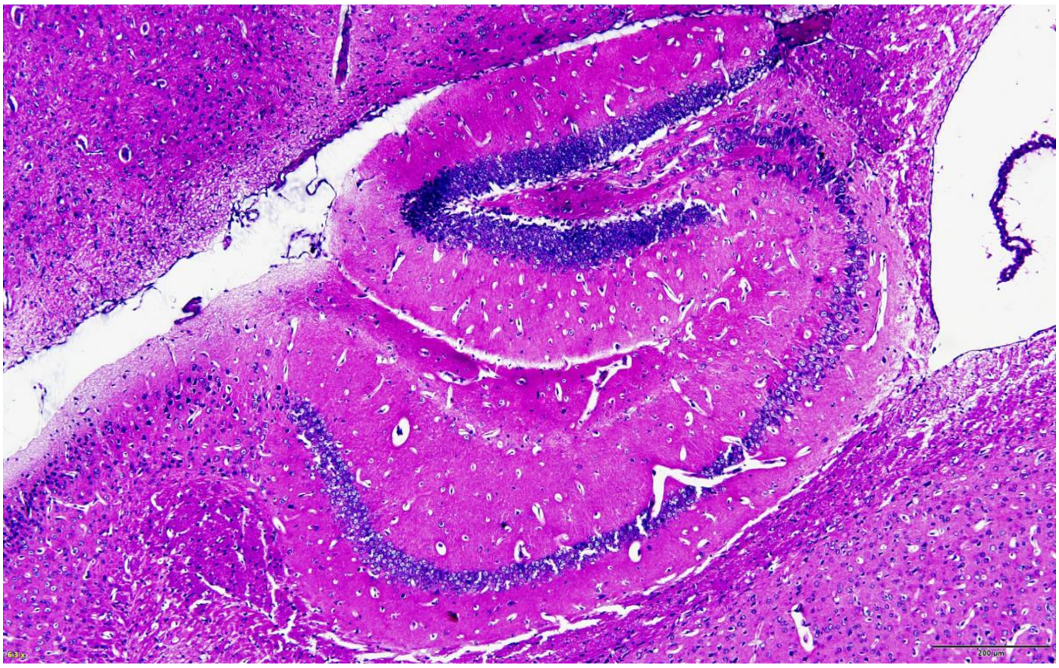

6F. Region of the hippocampus

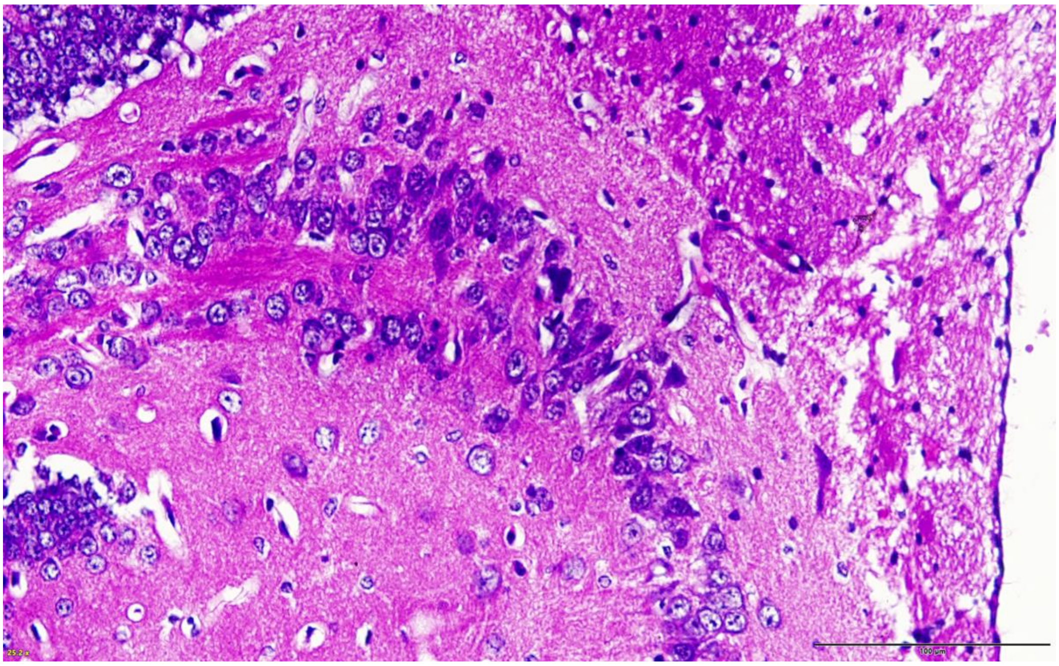

6F. Region of the hippocampus (black box)

Artesunate (Art) treated

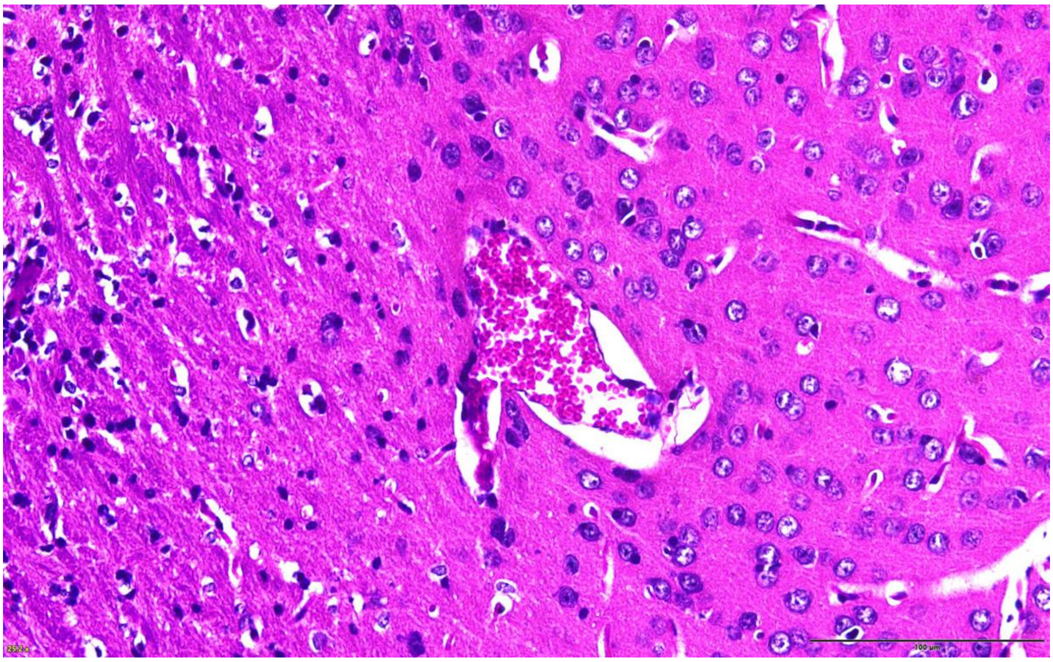

6G. Blood vessels

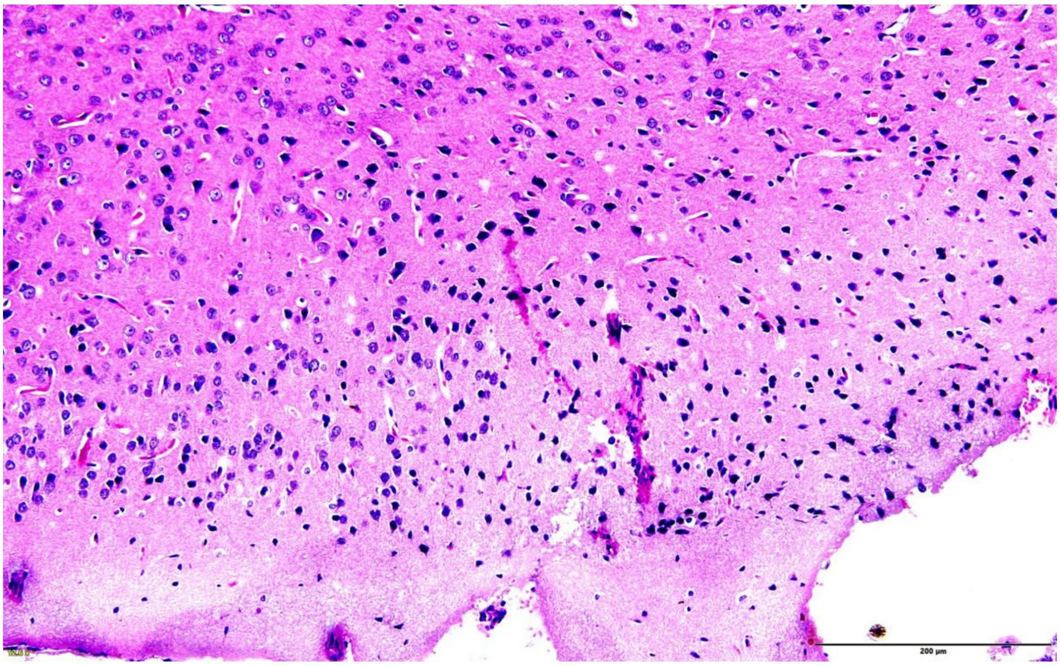

6H. Brain hemorrhage

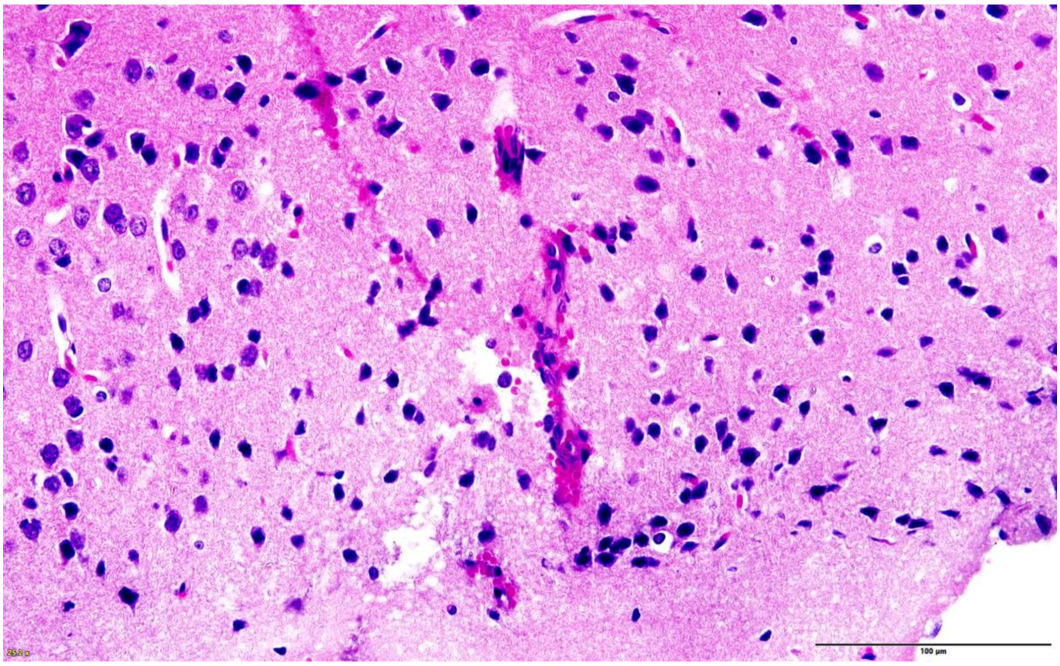

6H. Brain hemorrhage (black box)

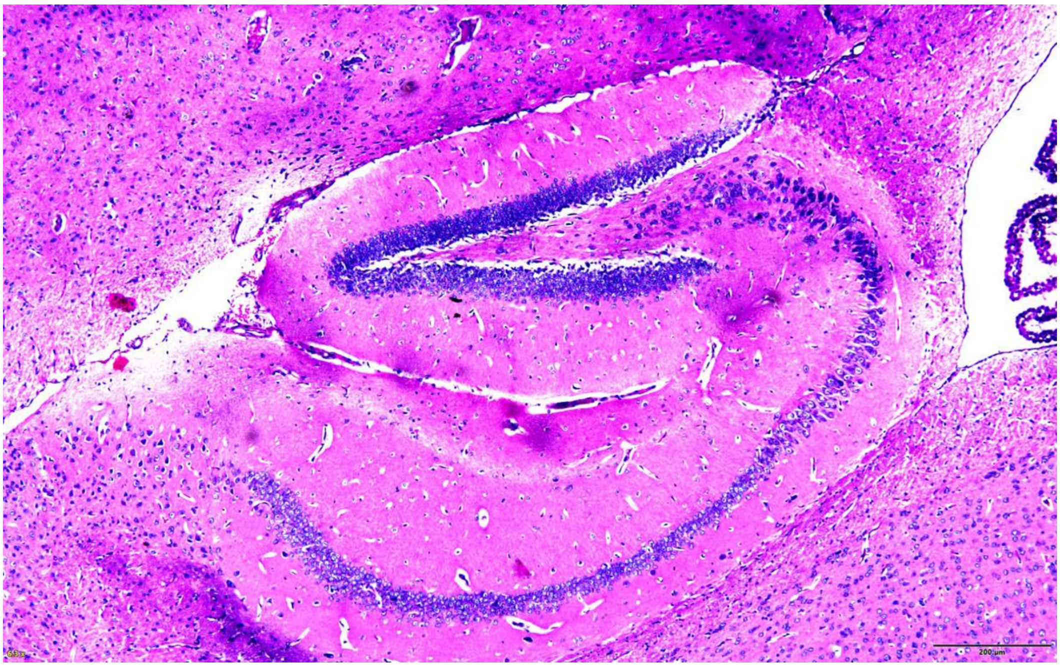

6I. Region of the hippocampus

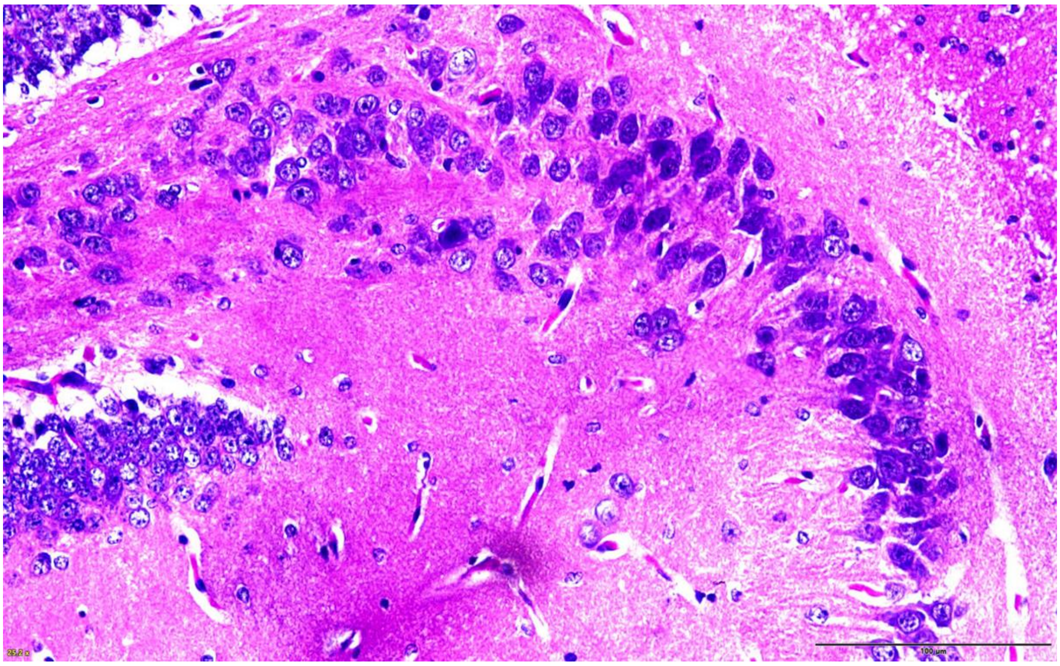

6I. Region of the hippocampus (black box)

Five flower group (FFR) treated

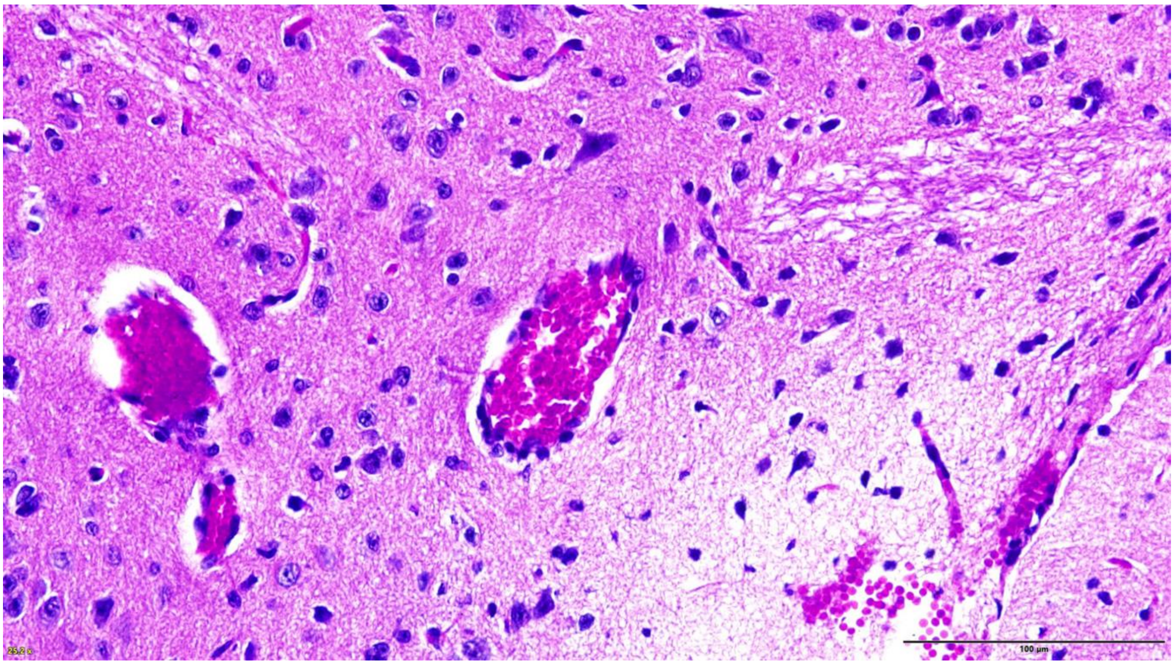

6J. Blood vessels

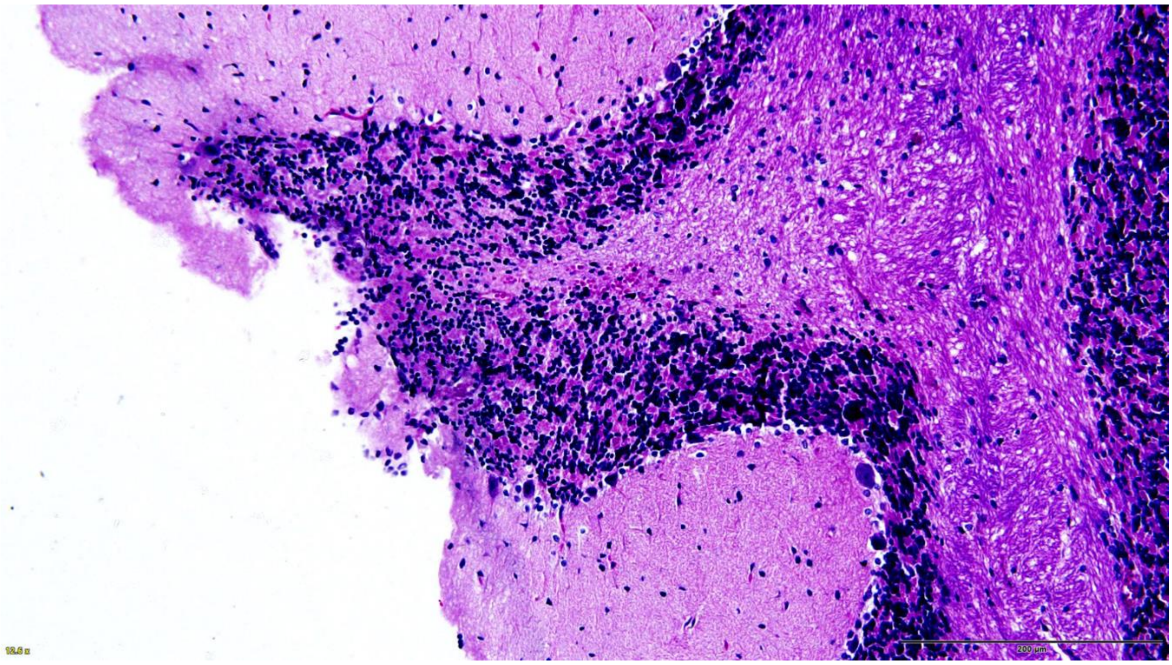

6K. Brain hemorrhage

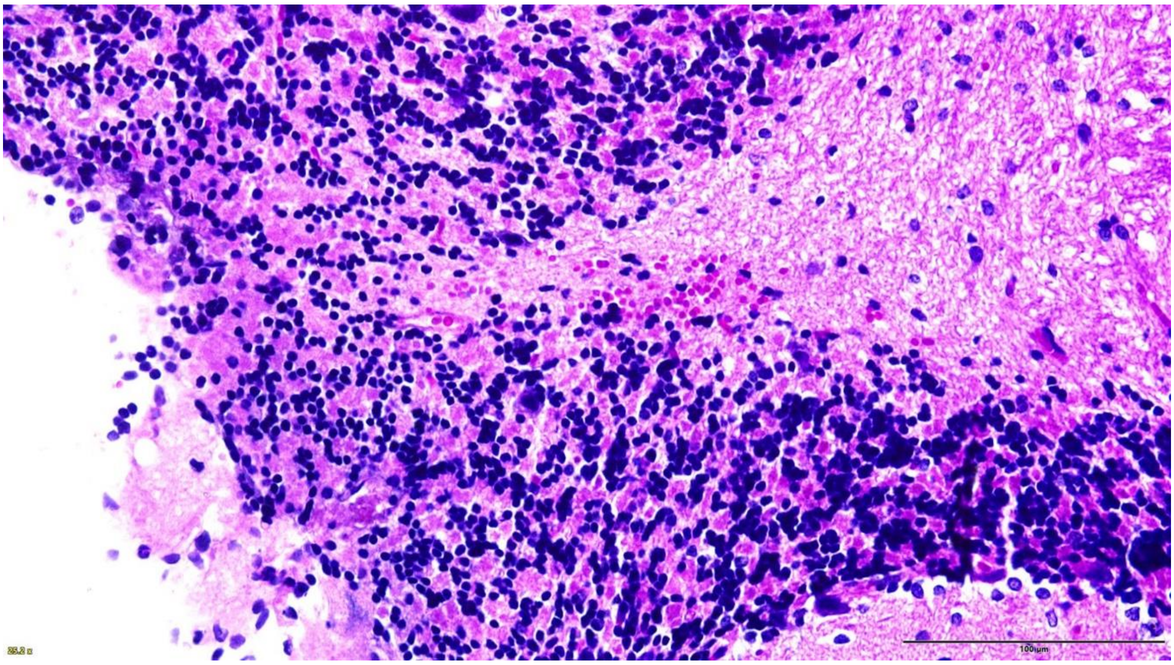

6K. Brain hemorrhage (black box)

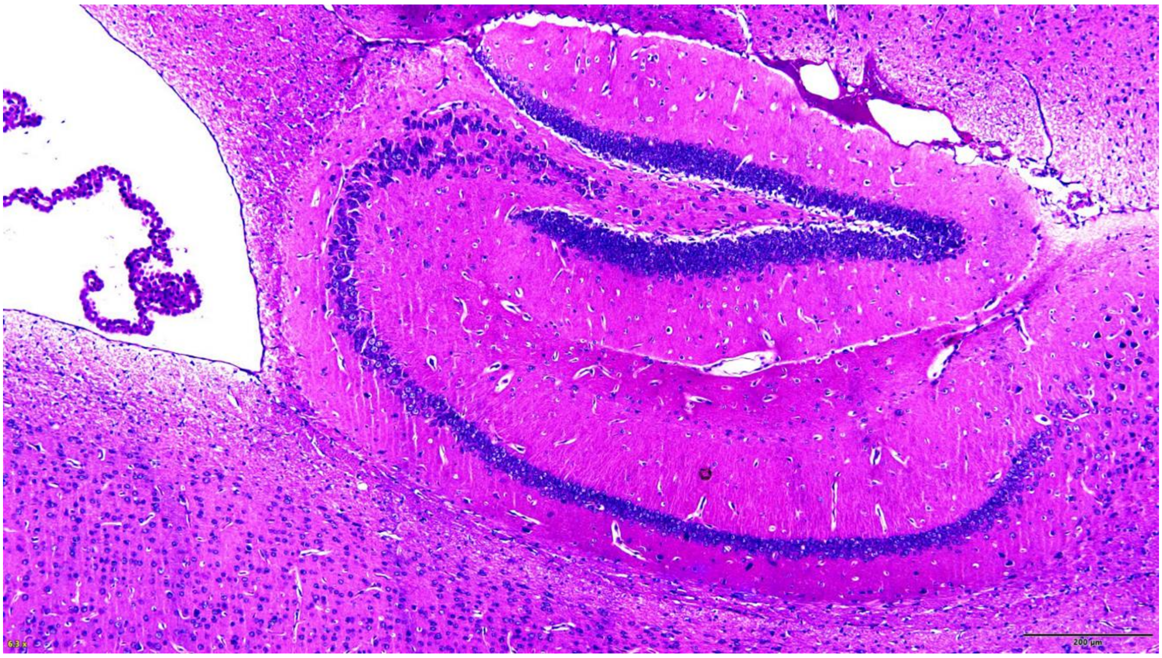

6L. Region of the hippocampus

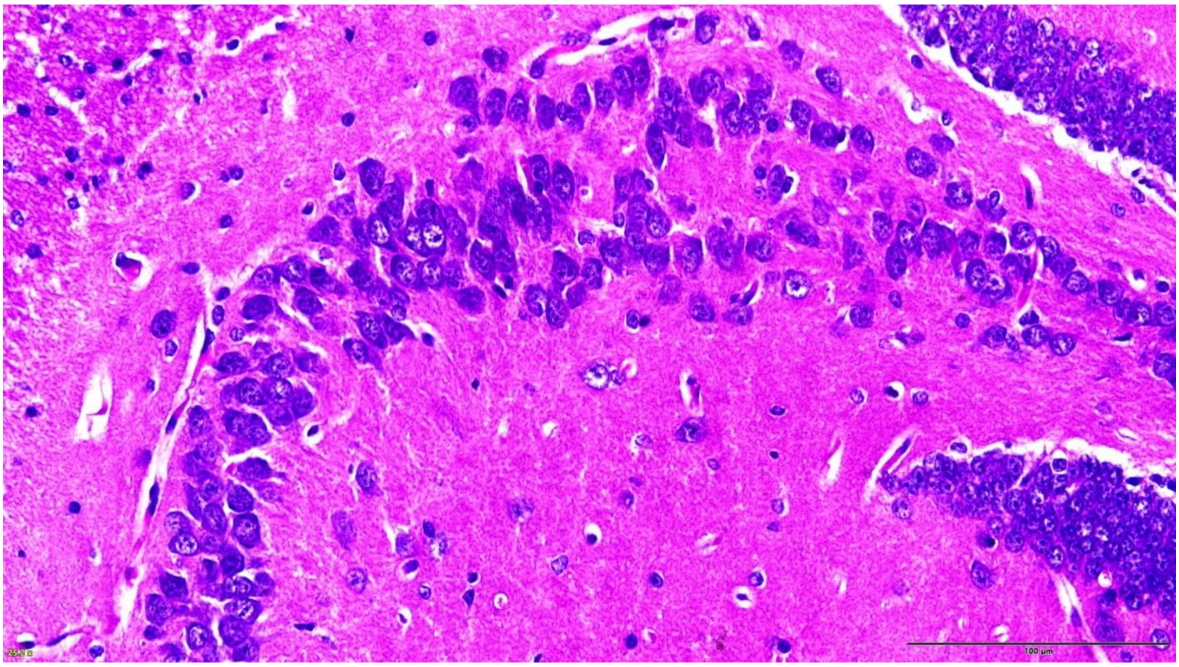

6L. Region of the hippocampus (black box)

Combination of artesunate with the Five-Flower Remedy

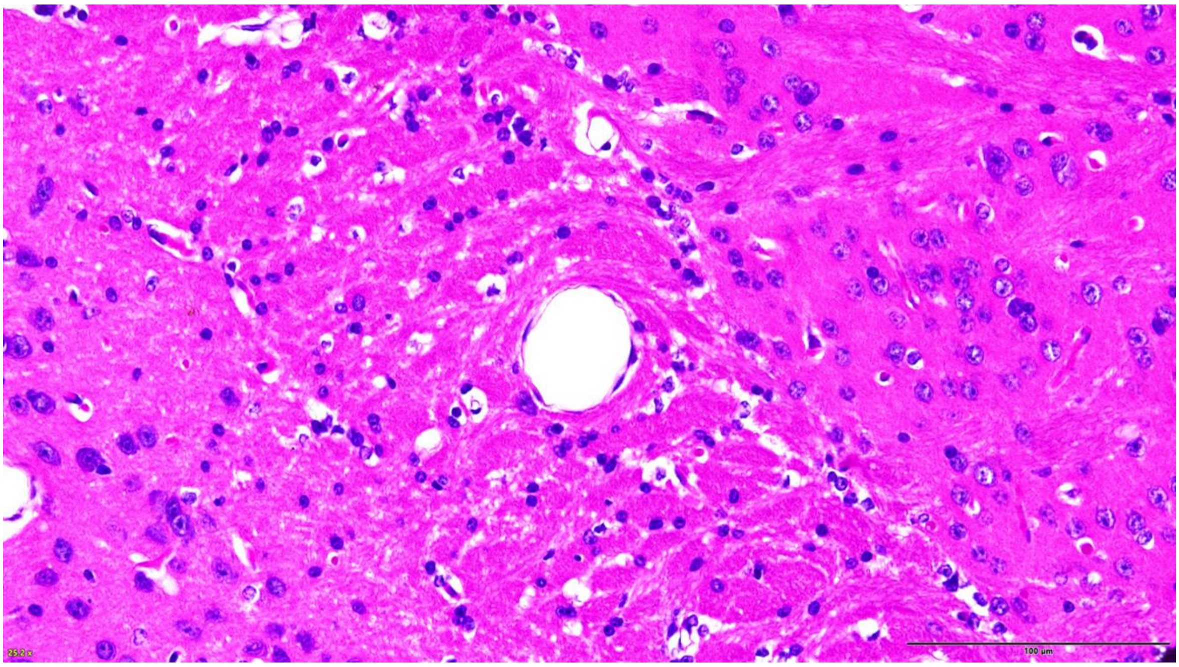

6M. Blood vessels

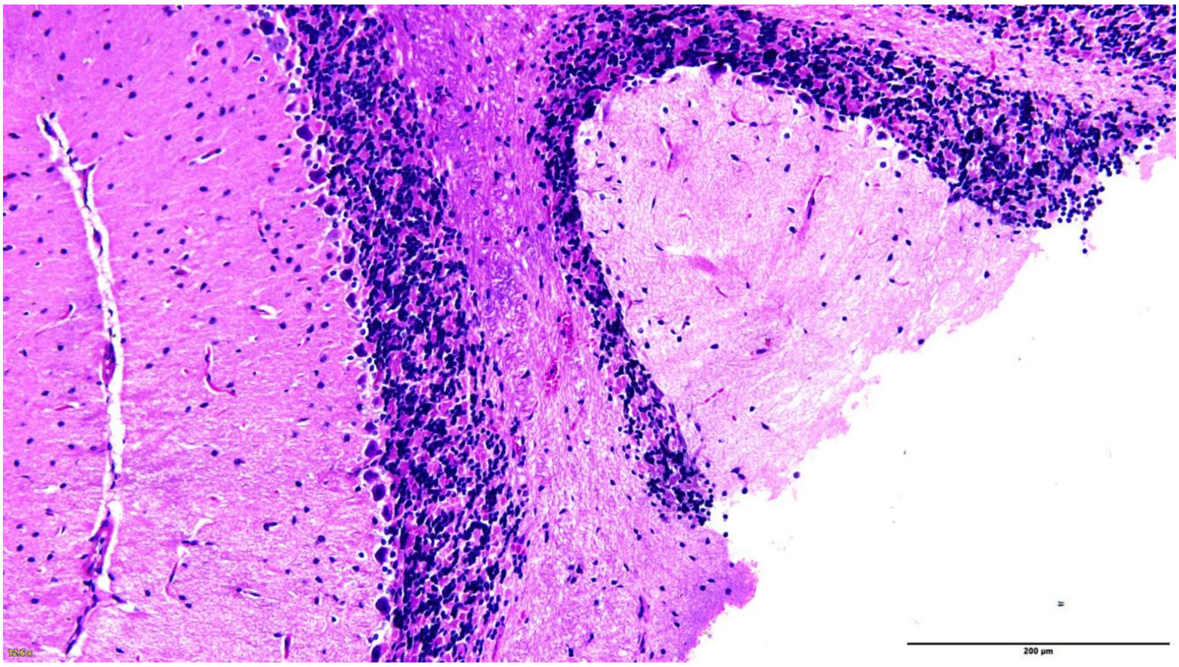

6N. Brain hemorrhage

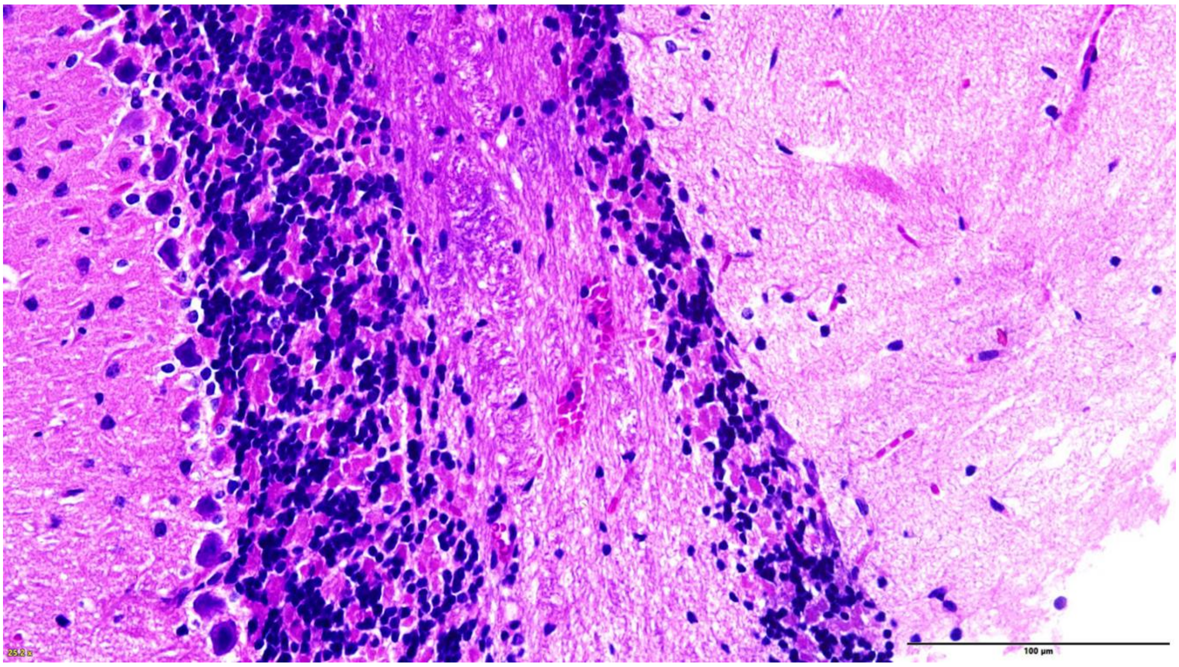

6N. Brain hemorrhage (black box)

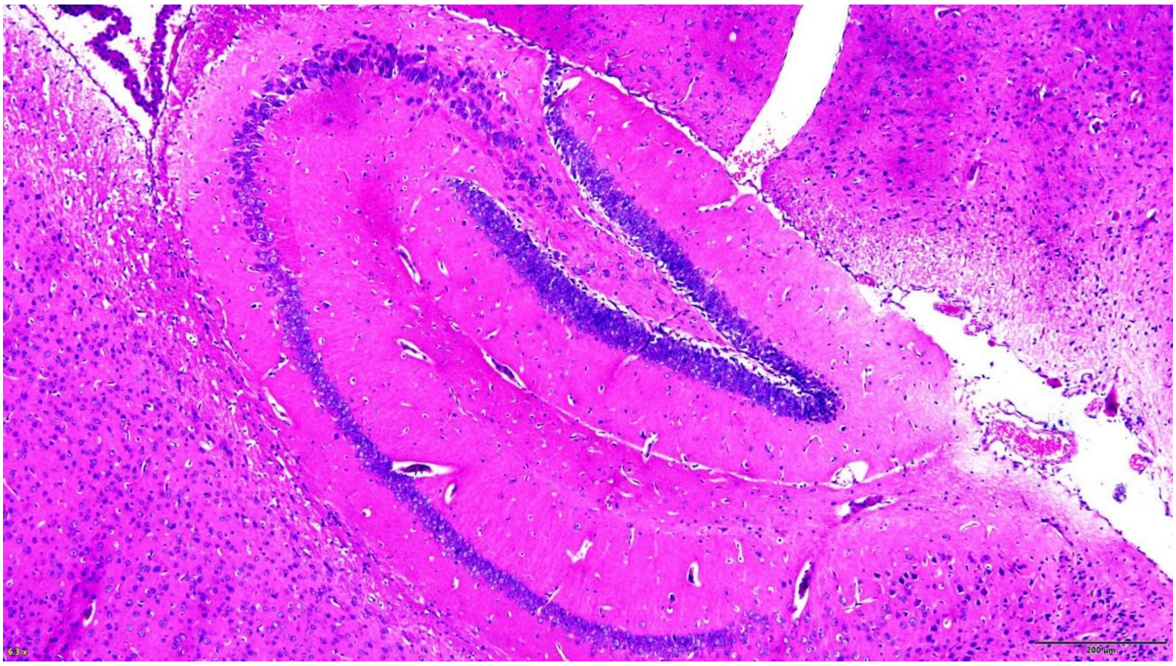

6O. Region of the hippocampus

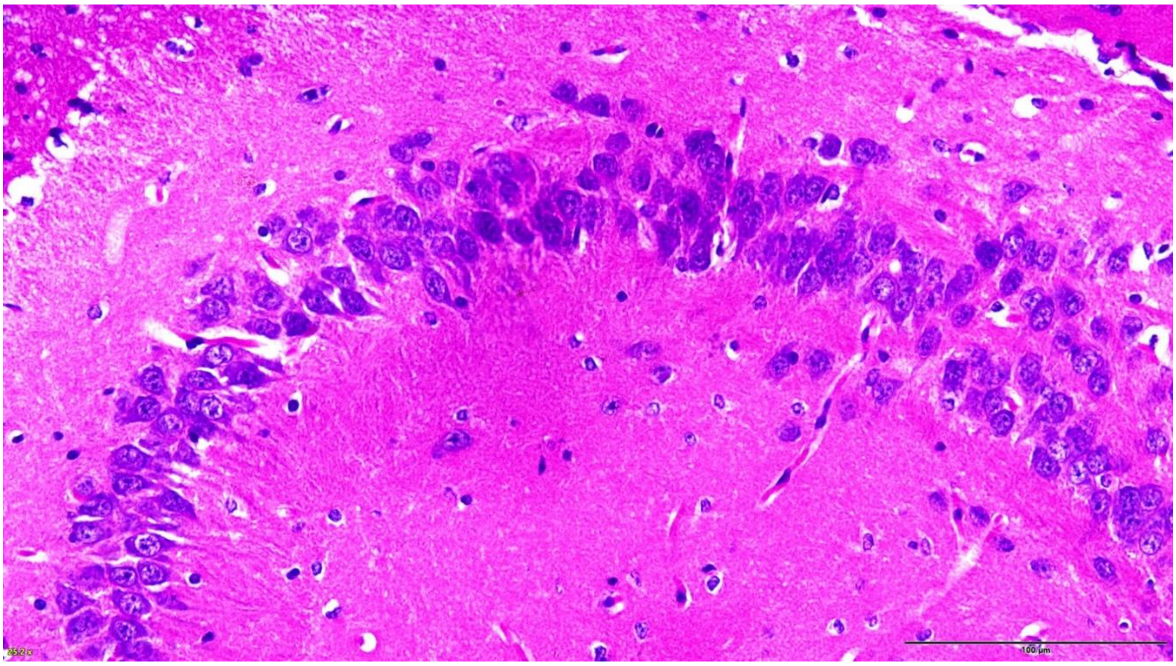

6O. Region of the hippocampus (black box)
