# Supplementary material for: Antimalarial and neuroprotective effects of ethanolic extracts of the five-flower remedy in an experimental cerebral malaria model
Source: PLoS One. 2025 Sep 2;20(9):e0330880. doi: 10.1371/journal.pone.0330880 (PMC12404382; doi:10.1371/journal.pone.0330880)
Supplement: S3 File — (PDF) [file pone.0330880.s003.pdf]

Supporting information file 3

Melt curve data for qPCR

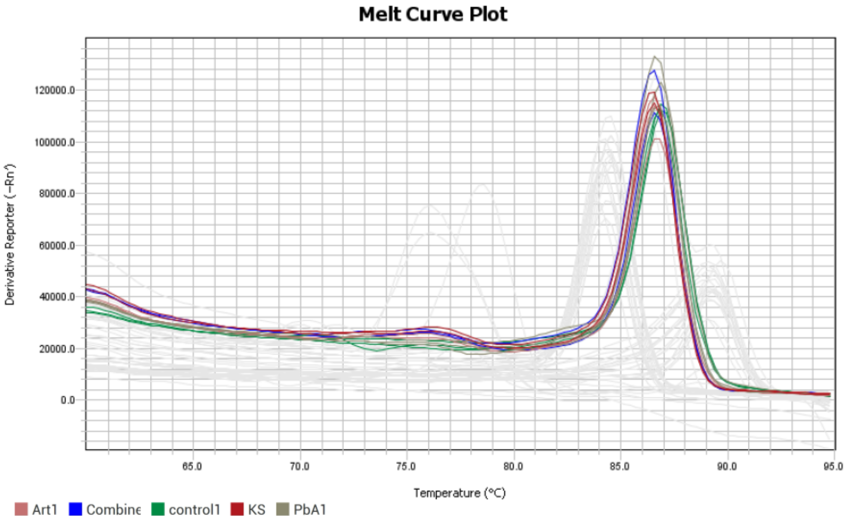

TNF-α melt curve plot

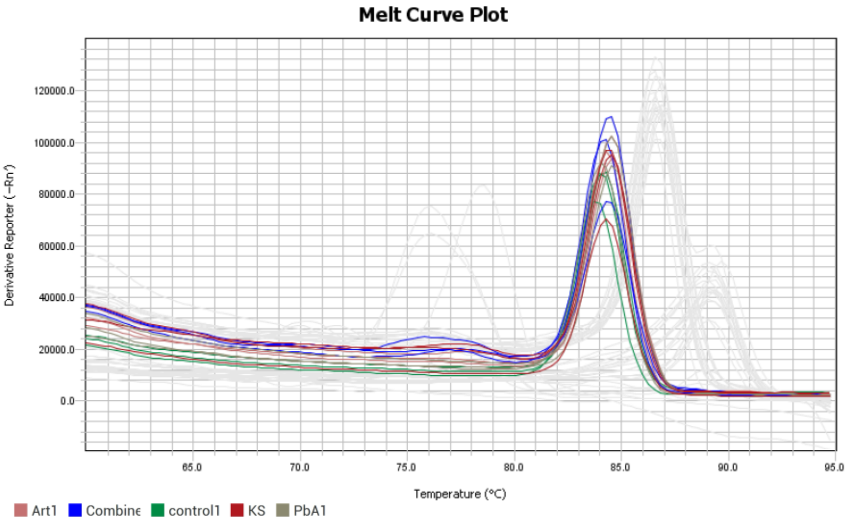

IL-1β melt curve plot

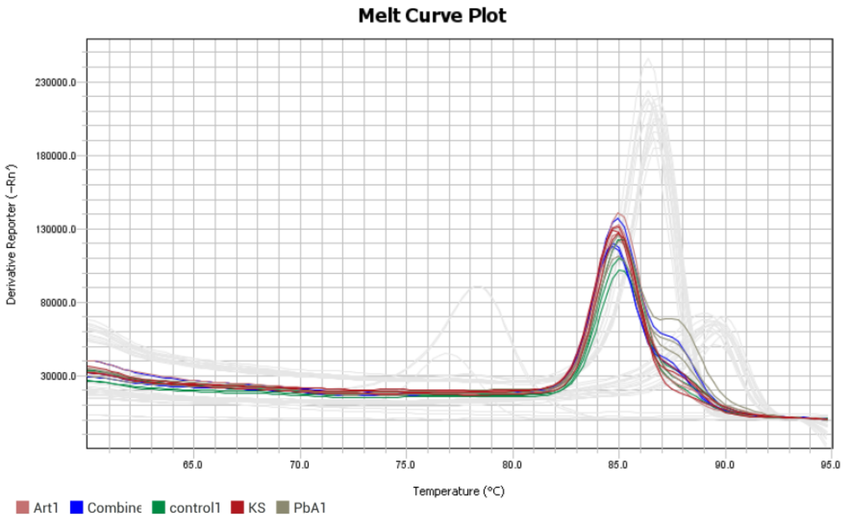

BDNF melt curve plot

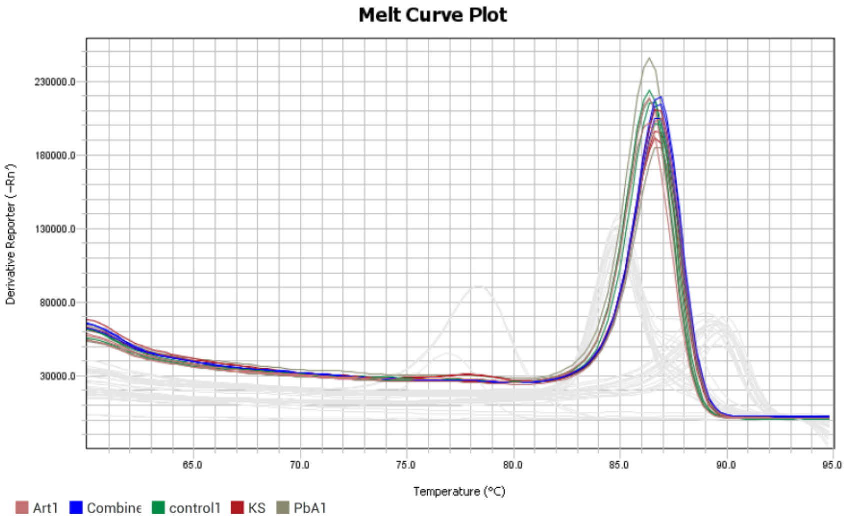

Trk B melt curve plot
