# Supplementary material for: Antimalarial and neuroprotective effects of ethanolic extracts of the five-flower remedy in an experimental cerebral malaria model
Source: PLoS One. 2025 Sep 2;20(9):e0330880. doi: 10.1371/journal.pone.0330880 (PMC12404382; doi:10.1371/journal.pone.0330880)
Supplement: Attachment — Submitted filename: Reviewer Comments to the Author.docx [file pone.0330880.s004.docx]

**Reviewer Comments to the Author**

Title: Antimalarial and neuroprotective effects of ethanolic extracts of the five-flower remedy in an experimental cerebral malaria model

**General Evaluation:**

This manuscript investigates the antimalarial and neuroprotective effects of the five-flower remedy (FFR), a traditional Thai herbal formulation, in a mouse model of cerebral malaria (CM). The authors provide compelling evidence that FFR, especially in combination with artesunate, can significantly reduce parasitemia, improve neurological function, and reduce neuroinflammation and neuronal damage. The study is timely, methodologically sound, and presents novel findings that may contribute to the development of adjunctive therapies for cerebral malaria. The manuscript is well written but it needs clarification in some points before being further considered for publication.

**Recommendation: Minor Revisions**

Major Strengths:

- The study addresses an important gap in cerebral malaria therapy, focusing on neuroprotective outcomes.
- The experimental design is well-structured, with appropriate control and treatment groups.
- The use of behavioral (RMCBS, NOR), histological, and molecular endpoints provides robust support for the findings.
- Results are clearly presented and discussed in a logical and contextualized manner.

**Specific Comments:**

**Introduction**

1. Consider summarizing FFR’s pharmacological background more concisely.
2. Clarify the novelty of the study—emphasize that this is the first in vivo assessment of FFR in CM.

**Materials and Methods**

1. At Plant preparation and extraction and Dosing of CM model, please explain the rationale behind the choice of 600 mg/kg FFR dose.
2. Clarify whether randomization and blinding were applied during group assignment and outcome assessment.
3. At the part of Dosing and grouping of CM model, why did the researcher start treatment on day 6-12, while behavioral changes were monitored on day 4?

**Results**

1. Should include Ct or melt curve data for qPCR in supplementary files to support gene expression results.
2. Please improve image resolution for histology figures and change the color of the arrowhead at Fig 6.

**Discussion**

1. Consider discussing the possibility of drug-herb interactions with artesunate.
2. Avoid redundancy—some background material from the introduction is repeated.
3. Recommend mentioning study limitations and suggesting future directions (e.g., mechanistic studies, dose-response, clinical evaluation).

**Conclusion**

- Strong conclusion supported by data.

**Final Verdict: Minor Revisions**

I recommend acceptance after minor revisions addressing methodological clarification and figure enhancement.
